# Characterization of the kidney transcriptome of the South American olive mouse *Abrothrix olivacea*

**DOI:** 10.1186/1471-2164-15-446

**Published:** 2014-06-08

**Authors:** Facundo M Giorello, Matias Feijoo, Guillermo D’Elía, Lourdes Valdez, Juan C Opazo, Valeria Varas, Daniel E Naya, Enrique P Lessa

**Affiliations:** Departamento de Ecología y Evolución, Facultad de Ciencias, Universidad de la República, Montevideo, Uruguay; Instituto de Ciencias Ambientales y Evolutivas, Universidad Austral de Chile, Valdivia, Chile

**Keywords:** *Abrothrix olivacea*, Abrotrichini, Cricetidae, Sigmodontinae, Muroidea, RNA-Seq, Gene expression, *De novo* assembly, Normalization methods

## Abstract

**Background:**

The olive mouse *Abrothrix olivacea* is a cricetid rodent of the subfamily Sigmodontinae that inhabits a wide range of contrasting environments in southern South America, from aridlands to temperate rainforests. Along its distribution, it presents different geographic forms that make the olive mouse a good focal case for the study of geographical variation in response to environmental variation. We chose to characterize the kidney transcriptome because this organ has been shown to be associated with multiple physiological processes, including water reabsorption.

**Results:**

Transcriptomes of thirteen kidneys from individuals from Argentina and Chile were sequenced using Illumina technology in order to obtain a kidney reference transcriptome. After combining the reads produced for each sample, we explored three assembly strategies to obtain the best reconstruction of transcripts, TrinityNorm and DigiNorm, which include its own normalization algorithms for redundant reads removal, and Multireads, which simply consist on the assembly of the joined reads. We found that Multireads strategy produces a less fragmented assembly than normalization algorithms but recovers fewer number of genes. In general, about 15000 genes were annotated, of which almost half had at least one coding sequence reconstructed at 99% of its length. We also built a list of highly expressed genes, of which several are involved in water conservation under laboratory conditions using mouse models.

**Conclusion:**

Based on our assembly results, Trinity's *in silico* normalization is the best algorithm in terms of cost-benefit returns; however, our results also indicate that normalization should be avoided if complete or nearly complete coding sequences of genes are desired. Given that this work is the first to characterize the transcriptome of any member of Sigmodontinae, a subfamily of cricetid rodents with about 400 living species, it will provide valuable resources for future ecological and evolutionary genomic analyses.

**Electronic supplementary material:**

The online version of this article (doi:10.1186/1471-2164-15-446) contains supplementary material, which is available to authorized users.

## Background

The olive mouse *Abrothrix olivacea* [1] is a cricetid rodent of the subfamily Sigmodontinae, one of the largest mammalian subfamilies with about 400 species and 86 living genera [2, 3]. The olive mouse is distributed along Chile and Argentinean Patagonia, from 18ºS to 55ºS latitude [4], extending for over 1000 km latitudinally, and encompassing a great variety of environments: coastal deserts in the north, Mediterranean scrubs in central Chile, Valdivian and Magallanic forests through the south of Chile and Argentina and Patagonian steppe towards the Atlantic coast. *A. olivacea* must withstand the arid Chilean north and the Patagonia steppe, as well as the Valdivian rain forest with 2700 mm or more of annual rainfall [5]. Given the striking biotic and abiotic differences among these environments, differences in thermoregulation and osmoregulation, among other physiological traits, are expected to occur. Higher tolerance to water shortage in populations from xeric habitat has already been demonstrated [6]. On the basis of variation in morphology, coloration patterns, and more recently DNA sequence data [4, 7], many *A. olivacea* subspecies have been described and at least two phylogeographic breaks have been found along its distribution [8]. All these characteristics make *A. olivacea* a good focal case for the study of geographical variation in response to environmental variation.

High-throughput sequencing (HTS) [9] has a wide range of applications, from clinical [10] to functional studies in genomics [11], molecular ecology [12], and microbial diversity [13]. Recently, HTS has also been used to characterize transcriptomes of a growing number of non-model species (e.g. [14–17]). RNA-seq is a cost-effective way to obtain large amounts of coding sequences and information about gene expression levels [18]. The goal of covering entire genome or transcriptomes, along with the reduction of the HTS costs [9], has motivated digital normalization strategies [19] to systematize the increasing but uneven coverage in shotgun sequencing datasets. Normalization methods estimate the read abundance, regardless of a reference, using the k-mer median abundance of that read and then decides whether to reject or accept it based on the chosen coverage value [19, 20]. In this manner, normalization algorithms remove redundant reads but also greatly reduce the total number of k-mers by discarding the majority of the erroneous ones. For example, with a sequencing base error rate of 1bp per 100 bp sequenced [9], k erroneous k-mers will be produced, being k equal to k-mers size. This data and error reduction notably decreases the computational requirements for *de novo* assembly.

In this study, we adopted paired-end Illumina sequencing to characterize the kidney transcriptome of *A. olivacea.* We chose kidney because of its association with multiple physiological processes, including water conservation [21] and nutrition [22]. This transcriptome will serve as a reference for comparative studies of geographical variation within this species, as well as for other studies on the diverse sigmodontine rodents. More than 800 million (M) reads were generated for 13 kidney transcriptomes of individuals sampled across Chile and Argentina*.* We explored various normalization strategies in order to obtain the best transcripts reconstruction and identify the most expressed genes. This is the first report of a sigmodontine transcriptome.

## Results

### Transcriptome sequencing and assembly

Transcriptome sequencing of 13 libraries using Illumina yielded a total of ~ 87 Gb of data, formed by ~430 M of paired reads with an average length of 101 bp (Additional file 1: Table S1). Trimming of low quality bases from the 3' end, prior to Trinity [23] *de novo* assembly, reduced average read length to 83 bp. The number of reconstructed contigs per library ranged from 62,499 to 120,209; with average length ranging from 972 to 1174 bp and median from 488 to 585 bp (Table 1). Detailed results for each library are shown in Additional file 1: Table S2.

**Table 1:** **Main assembly metrics for the three assembly strategies and individual libraries**

	Range (of individual libraries) ^a^		Multireads	TrinityNorm	DigiNorm
	min	max			
**Reads**	27041064	42477318	430525978	21757448	50557782
**Total contigs**	62499	120209	275903	277014	362916
**Max contig length**	9942	15496	20648	19625	15961
**Min contig length**	201	201	201	201	201
**Average length**	972	1174	1060	1210	1269
**Median length**	488	585	443	575	696
**Running time (hours)**	n/a	n/a	94 (12 threads)	10 (12 threads)	19 (12 threads)
**Normalization time (hours)**	n/a	n/a	n/a	14 (1 thread)	9 (1 thread)

^a^“Range (of individual libraries)” shows for each row the maximum and minimum value found among the 13 individual libraries of kidney transcriptome of the olive mouse *Abrothrix olivacea*.

To obtain a good reference transcriptome, we also explored three strategies: (i) combining reads of all libraries (Multireads), (ii) Trinity's *in silico* normalization (TrinityNorm) [20], and (iii) digital normalization (DigiNorm) [19]. The last two strategies involve, in order to improve assembly efficiency from high coverage sequencing datasets, the deletion of redundant reads, ideally without harming the quality of the final reconstructed genes. Of these two, TrinityNorm was more severe than DigiNorm in reducing the total number of paired-ends reads from ~430 M to ~22 M vs. ~50 M (Table 1). Meanwhile, digital normalization was faster than *in silico* Trinity normalization: 9 hours vs. 14 hours.

As expected, the Multireads strategy led to a far more time consuming and computationally demanding assembly than either of the normalization methods, being five and over nine times slower than the assembly from DigiNorm and Trinity, respectively (Table 1). Also, the average and median lengths of reconstructed contigs from the Multireads data set were smaller than the assembled contigs from normalized reads, with 1,060 and 443 bp for mulitreads, 1,210 and 575 bp for TrinityNorm, and 1,269 and 696 bp for DigiNorm. These results are consistent with the distribution of the contigs, where almost half (46%) of the reconstructed contigs from the Multireads strategy were between 200 and 400 bp (Additional file 1: Table S3). On the other hand, the Multireads strategy reconstructed the longest contigs (Additional file 1: Table S3) with 4,212 above 6,400 bp. TrinityNorm and Diginorm reconstructed only 3,073 and 2,726 of contigs above this length, respectively.

The two normalization strategies produced similar assembly results in terms of average and median length of contigs, with a small advantage for DigiNorm values, but they significantly differed in the number of contigs assembled, DigiNorm assembled 85,902 more contigs than TrinityNorm and 87,013 more than the Multireads strategy (Table 1).

### Gene annotation and evaluation of reconstructed coding sequences

Annotation was based on BLASTX searches against: (i) OMA browser mouse protein database, which contains the protein isoforms of *Mus musculus* genes [24] and (ii) NCBI non-redundant vertebrate protein database. For the two databases the same e-value threshold of 1e-10 was set. For the Multireads, TrinityNorm and DigiNorm strategies, each assembled transcript was also analyzed through the Pfam database [25] using HMMER [26, 27] for proteins domain identification. A file summarizing the Pfam and BLASTX results for each of the three strategies is available as Additional file 2.

The maximum number of mouse genes annotated within a particular library was 12,988 from the significant hits of 55,332 contigs of the 120,209 assembled (Table 2 and specimen PPA 443 library in Additional file 1: Table S2 and Table S4a). The union of the 13 individual BLASTX runs only added 1,630 significant hits (14,618 in total), indicating the high level of redundant information across libraries. On the other hand, when using the extensive non-redundant vertebrate database as reference, the maximum number of contigs annotated within a single library was 58,404, 3072 contigs more than with the OMA database (Additional file 1: Table S4b). Detailed results for each library are shown in Additional file 1: Table S4. Hereafter we present the results based only on mouse proteins from OMA. This database allow us to count the number of genes and their corresponding reconstructed coding sequences (CDS) and obtain an upper bound estimation of genes orthologous with mouse.

**Table 2:** **Gene annotation and coding sequences reconstruction using BLASTX to OMA browser mouse protein database**

	Minimum % of CDS reconstructed	Range (of individual libraries)		DigiNorm	Multireads	TrinityNorm	Library union
		min	max				
**Genes**	99	3090 (3102)	5021 (5037)	5211 (5245)	7017 (7053)	5895 (5926)	7060 (7131)
**Contigs**		4371	7763	15815	14881	13671	n/a
**Genes**	90	4534 (4557)	6882 (6916)	7354 (7436)	9467 (9543)	8252 (8325)	9290 (9434)
**Contigs**		6861	11361	26904	22137	21825	n/a
**Genes**	80	5706 (5745)	8636 (8636)	8530 (8665)	10377 (10480)	9347 (9467)	10104 (10367)
**Contigs**		5422	14129	36814	26745	28270	n/a
**Genes**	50	8025 (8138)	9905 (10089)	12261 (12610)	12587 (12818)	12498 (12769)	11874 (12544)
**Contigs**		14168	25674	76156	40607	51140	n/a
**Total genes**		11564 (11941)	12988 (13434)	15095 (15717)	14788(15204)	15077 (15605)	14618 (15772)
**Total contigs**		32934	55332	157390	70380	100786	n/a

The first value indicates the number of mouse genes found (for which at least one coding sequence was reconstructed). Values in parenthesis are the number of distinct coding sequences reconstructed at each level. The row corresponding to “contigs” indicates the number of contigs that reconstructed coding sequences (CDS) at each level.

Of the 14,618 mouse genes annotated through the union of all libraries, almost one half (7,060) had at least one putative CDS reconstructed at > 99%, 9,290 at > 90%, and 10,104 at > 80%, of the total expected length. More importantly, in total, 9,434 distinct mouse isoforms of the 24,338 (~39%) available at OMA browser were almost fully reconstructed (>90%) for *A. olivacea* (Table 2).

**Figure 1:**
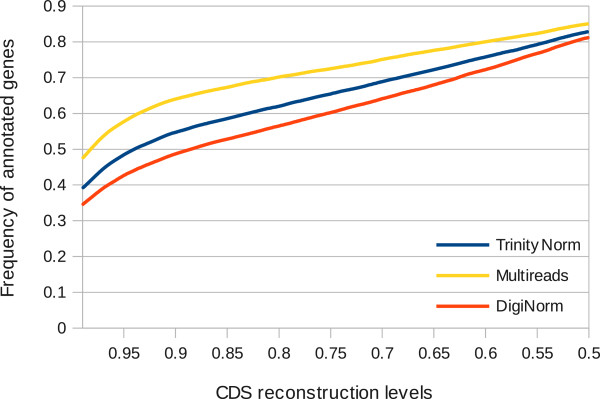
**Frequency of genes annotated for** ***Abrothrix olivacea*** **at corresponding CDS reconstruction levels.** Frequency of genes annotated per total number of genes were calculated for each strategy (see text) and for the corresponding mouse coding sequences (CDS) reconstruction level. The CDS reconstruction intervals are of 0.01 percent. Only the largest reconstructed CDS for each gene are taken into account.

Among the three strategies carried out to obtain a reference transcriptome, Multireads reached the lowest number of mouse genes, 14,788; meanwhile TriniNorm and DigiNorm, reached 15,077 and 15,095 respectively. Despite having found the lowest number of genes, the Multireads strategy performed best at reconstructing coding sequences, obtaining similar values to those gathered through the union of the single libraries, with 7053 distinct mouse coding sequences reconstructed at > 99%, 9,543 at > 90%, and 10,480 at > 80% (Table 2). With regard to genes, of the 14,788 annotated by the Multireads alternative, 47% had at least one CDS fully (>99%) reconstructed, clearly surpassing the 39% and 34% of TrinityNorm and DigiNorm respectively (Figure 1). Between the two normalization strategies, TrinityNorm outperformed DigiNorm at each reconstruction level in terms of numbers of mouse genes found and percentage of coding sequence reconstructed (Table 2 and Figure 1). On the other hand, the assembly from DigiNorm had more contigs at each level of reconstruction and also more contigs with at least one Pfam domain, followed by TriniNorm and the Multireads strategies (Table 2 and Additional file 1: Table S5). This is expected if those contigs represent distinct fragments of the same coding sequence or if they are isotigs (overlapping contigs) representing (ideally) distinct isoforms. However, when the number of potential isoforms from Trinity assembly were inferred and counted (see methods), the average number of alternative reconstructions per contig was 4.9 for DigiNorm and only 2.9 for the Multireads strategy (data not shown). Thus, those contigs are alternative reconstruction (isotigs) representing (possibly) isoforms and not subfragments of a given reference.

### Functional annotation

For the functional annotation of the transcriptome, we selected the genes found by TrinityNorm. This strategy was the one with the best tradeoff between CDS reconstructed, genes found, and computational speed. To this end, the Database for Annotation, Visualization and Integrated Discovery (DAVID) [28], was used to classify them with Gene Ontology (GO) terms.

**Figure 2:**
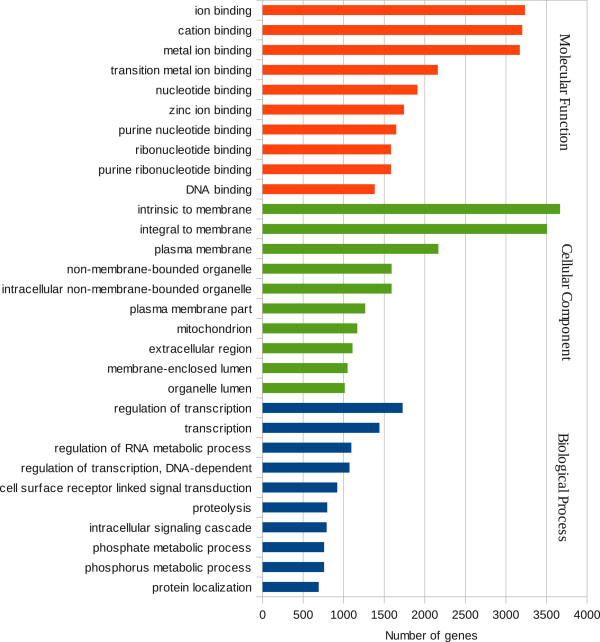
**Gene Ontology classification of genes found by TrinityNorm strategy.** Results are summarized in three main categories: Biological process, Molecular function, and Celullar component.

For the 15,077 genes found by TrinityNorm strategy, 9,793 GO terms were categorized in Biological Processes, 9,486 in Molecular function and 8,978 in Cellular Components. Most genes at Biological Processes belong either to “Regulation of transcription” (1,726), “Transcription” (1,441) and to “Regulation of RNA metabolic process” (1,093) (Figure 2). Likewise, the Molecular Function category subdivided annotated sequences into “ion binding” (3,234), “cation binding” (3,201), and “metal ion binding” (3,172) as the most represented (Figure 2). Within the category Cellular Component, the three principal groups were: “intrinsic to membrane” (3,667), “integral to membrane” (3,506) and “plasma membrane” (2,167) (Figure 2).

### The most expressed genes

To determine the most expressed genes in the *A. olivacea* kidney transcriptome, TPM (*Transcripts Per Million*) expression values were calculated for each single library with RSEM software [29]. For this purpose, a set with 5% of most expressed genes (~600 genes) for each of the 13 transcriptomes was identified; these were cross searched to identify those genes common to all libraries. Two hundred eighty-three genes resulted to be present in all libraries (Additional file 3: Table S5). The average TPM values ranged from 333 to 17,798 (Additional file 3: Table S5). Five genes that showed the highest average TPM values were: predicted gene 4076 (possibly a NADH-ubiquinone oxidoreductase) (ENSMUSG00000096449), glutathione peroxidase 3 (ENSMUSG00000018339), ferritin heavy chain 1 (ENSMUSG00000024661), hemoglobin beta adult major chain (ENSMUSG00000052305), and phosphoenolpyruvate carboxykinase 1 cytosolic (ENSMUG000000027513) (Additional file 3: Table S5).

GO terms for the 283 genes obtained from DAVID showed that the most enriched terms among the three domains using the TrinityNorm genes list as background were: “hydrogen ion transporting ATP synthase activity, rotational mechanism” (17.4 Fold Enrichment, domain: molecular function), “proton-transporting ATP synthase complex” (25.4 Fold Enrichment, cellular component) and “mitochondrial ATP synthesis coupled electron transport” (25.3 Fold Enrichment, biological process) (Additional file 3: Table S6).

Subsequently, these 283 genes were cross-checked with a list obtained from Pradervand *et al.* [30], who enumerated the most expressed genes in the distal part of the mouse renal tubule using microarrays. Seventeen genes resulted to be in common (Additional file 3: Table S5 in bold): two transcription factors, one small GTPase, eight transporters and channels, and six cytoskeleton-related genes. Among these genes are Aquaporin 1 (*Aqp1*), Ras-related protein Rab-7a (*Rab7*), Sodium/potassium-transporting ATPase gamma chain (*Fxyd2*), Voltage-dependent anion channel 1 (*Vdac1*), and Guanine nucleotide binding protein, alpha stimulating (*Gnas*).

## Discussion

The subfamily Sigmodontinae includes about 400 species and 86 living genera [2, 3]. Of these, *A. olivacea* inhabits a wide range of contrasting environments and presents different geographic forms. In this work, 13 individuals were sampled in Argentina and Chile, covering both the arid Patagonian steppe and the wet Valdivian and Magellanic forests. More than 800 M reads were generated in what constitutes the first characterization of a sigmodontine transcriptome. In addition, we present a set of highly expressed genes of which some are possible candidates for ecological studies of the response of the species to environmental variation in water and dietary items availability.

### Transcriptome sequencing and assembly

As the cost of high-throughput sequencing falls and more cDNA sequences are generated, the importance of appropriate normalization strategies prior to contig assembling increases. In this study, we used two normalization strategies and compared their performance with a non-normalized (Multireads) alternative.

In terms of length and number of contigs assembled regardless of the strategy, our results were similar to those found in previous studies in which Trinity was used [31, 32]. Among the three strategies, if only contigs descriptive statistics are taken into account, the assembly after both normalization strategies clearly outperformed the Multireads approach. Normalizations not only showed the largest average and mean contig lengths but also ran considerably faster than the Multireads counterpart. This is a consequence of discarding reads that are considered to be redundant and the concomitant sequencing error removal. If transcriptome coverage is moderate, it is necessary to keep in mind that, as noted by Brown *et al.* [19], the memory requirements will be roughly the same, with or without normalization, due to limited removal of erroneous k-mers.

### Gene annotation and evaluation of reconstructed coding sequences

To evaluate the capacity of each strategy to assembly the *A. olivacea* transcriptome, we quantified the number of contigs that resulted to be homologs of mouse OMA proteins at different reconstruction levels. We found that the Multireads strategy performs the best for the > 99%, > 90 and > 80% of the total mouse CDS length and obtained the most similar values to those found through the union of the individual sets of contigs. Even though the Multireads strategy produced the assembly with smaller median and mean contig sizes, larger contigs were reconstructed (Additional file 1: Table S3), thus explaining the better results obtained for the CDS reconstruction. Therefore, studies that require complete reconstruction of coding sequences should avoid normalization; but obviously, when billions of reads are available for assembly, the Multireads approach becomes prohibitive and normalization is the only way to proceed. These results are consistent with those found by [19]; it seems that normalization generates a more fragmented assembly, at least when Trinity is used. Our results are consistent with the notion that fragmentation is a by-product of normalization, but also that normalization negatively impacts the completeness of the coding sequences reconstruction at all levels. For both normalization methods a k = 25 (k-mer) was set, and it is not clear why such fragmentation is produced.

If assembly capacity and computational requirements are considered, we consider that assembly from TrinityNorm has the best cost-benefit return. This strategy was second after Multireads in number of genes reconstructed for each category while using about 1/10 of the time. Moreover, a high number of putative isoforms were reconstructed, supported by the number of contigs reconstructed per category (Table 2) and the isotigs counted from Trinity assembly (see Results). Even though DigiNorm reached the largest number of isotigs and the highest number of genes, its performance at reconstructing full and almost full CDS was the worst and very similar to the best single library. Also, the assembly from DigiNorm was two times slower than that from TrinityNorm. The latter required more time for normalization but is capable of running multithreading and so outperforms DigiNorm.

Regardless of the strategy, a large number of homologous mouse genes were obtained in our study. According to microarray studies [33] *,* about 7600 genes are expressed in the kidneys of adult humans; meanwhile, recent HTS transcriptome studies found 15,369 for the baboon kidneys [34]. Clearly, in this study we were able to find almost the same number of genes (15,077 through TrinityNorm) that Spradling *et al.* [34] even though we used a very stringent e-value for the BLASTX analysis. Moreover, of those genes, 40 % had at least one full (>99%) CDS reconstructed. Given that, we reconstructed 8,325 distinct mouse isoforms out of 24,338 (~34%) for at least 90% of the total expected length, we established an important set of sequences, likely orthologous to mouse genes, which will be useful for future analyses of molecular evolution, population genomics, and phylogenetics.

### List of most expressed genes

The assessment of gene differential expression tends to be problematic for contigs with low counts [35]; therefore, a good strategy is establishing a set of highly expressed genes for directing efforts to study differential expression. In this work, 13 transcriptome libraries were used to identify the most expressed genes of the kidneys of *A. olivacea*. Some of them had already been described for model species, while many of the new ones have a clear relationship with renal function and could serve as potential candidates for future evolutionary and ecological genomic studies.

Seventeen of 283 most expressed genes found in this work were previously singled out by Pradervand *et al.* [30] using microarrays in the distal part of the mouse renal tubule. Although for some of these genes their precise function is not clear, for others knowledge on their function is reasonably good. For example, *Aqp1* is involved in water reabsorption at the apical and basolateral plasma membrane of the proximal tubule [36]; mutations in *Fxyd2* have been associated with renal hypomagnesemia-2 [37]; *Rab7*, as a Rab member, could be implicated in the transport, docking, and fusion of endocytotic vesicles [30], and finally, *Gnas* codifies the alpha subunit of heterotrimeric G proteins, which mediates the vasopressin receptor type 2 signaling after the binding with vasopressin, and ultimately increases water reabsorption in the collecting duct [36]. In our expression analysis, *Fxyd2* and *Aqp1* are among the top 10 and top 50 of the most expressed genes, respectively. The latter represents a good candidate gene for the study of differential responses to variation of environmental water availability.

Among the 266 remaining highly expressed genes, additional putative candidates associated with renal function were found; for example: i) kallikrein (*Klk1)* encodes a proteolytic protein which produces the kinin proteins, which may counteract the hydrosmotic effect of vasopressin [36]; ii) Uromodulin (*Umod*) encodes the most abundant protein in urine [38], and mouse knockouts for this gene have shown urine concentration problems [36]; iii) Glyoxylate reductase (*Grhpr*), an enzyme that catalyzes the reduction of glyoxylate to glycolate, is associated with a disorder that can cause nephrolithiasis (kidney stone), nephrocalcinosis, and renal failure [39, 40]; and iv) Sorbitol Dehydrogenase (*Sord*), along with Aldose reductase, are possibly involved in osmoregulation in the kidney [36, 41].

Regarding the GO-term classification, no important differences were found between the set of all genes and the 283 most expressed ones, except that an expected excess of mitochondrial related GO-terms was found among the latter (Additional file 3: Table S6). This enrichment is not surprising as the kidney is an energetically demanding organ [42, 43].

## Conclusion

In order to obtain the best-reconstructed transcripts from the kidney of the olive mouse *A. olivacea* on the basis of 13 individual libraries, we first explored three alternative assembling strategies. Results indicate that the Trinity's *in silico* normalization is the best algorithm in terms of cost-benefit return. We annotated more than 10,000 genes that were almost fully reconstructed, calculated their expression levels, and identified the most expressed ones. Various genes involved in water conservation in mouse models under laboratory conditions were reconstructed and showed high expression levels in *A. olivacea*, demonstrating the value of RNA-seq technology. Given that this work is the first to characterize the transcriptome of any member of Sigmodontinae, a subfamily of cricetid rodents with about 400 species, it will provide valuable resources for future ecological genomics and evolutionary analyses and will serve as assembly reference for a large number of species. In particular, it will facilitate the study of variation in levels of gene expression in the olive mouse and other sigmodontines that occupy a wide range of environmental conditions—from aridlands to temperate rainforests—in South America.

## Methods

### Data collection

Individuals were collected with Sherman traps from the following localities: Fundo San Martín, Los Ríos (n =4) and Sector Barrancoso, Aysén (n = 4) in Chile, and Gan Gan, Chubut (n =2) and Río Oro, Santa Cruz (n =3) in Argentina (further details in Additional file 1: Table S1). Kidneys were frozen in liquid nitrogen in the field immediately following euthanization. All steps involving live animals followed the recommendations of Sikes *et al.* [44].

### RNA extraction and library construction

For each individual, RNA extraction was conducted in one half of the kidney after a lengthwise cut. To this end, the RNeasy mini kit (Qiagen) was employed following recommendations of the manufacturer. RNA quantity and purity was assessed with NanoDrop 1000 Technologies spectrophotometer. RNA integrity was checked through electrophoresis in Formaldehyde-agarose 1,2% denaturing gels. Libraries were constructed and sequenced at Macrogen (Korea). Poly-A based mRNA enrichment method and paired-ends library preparation were done following the Illumina TruSeqTM RNA sample preparation kit, according to the instructions of the manufacturer. Library sequencing was performed on Illumina HiSeq 2000 platform.

### De novo transcriptome assembly

Assembly was carried out using default Trinity settings, after removing low quality reads, filtering adaptors and primers, and trimming the 3' ends of reads with a quality less than 24 (Q < 24) with FASTX-Toolkit (http://hannonlab.cshl.edu/fastx_toolkit/). Quality control was checked by FastQC (http://www.bioinformatics.babraham.ac.uk/projects/fastqc/). All assemblies were done on the same single node-machine with 256G memory and 4 Intel Xeon CPU E7-8837 (8 core) processors. In order to obtain a more complete set of the genes expressed, we pooled individuals from different points of the species distribution and analyzed three strategies: i) merge the reads of the 13 libraries (Multireads) ii) Trinity *in silico* read normalization (TrinityNorm v2013-08-15), and iii) digital normalization (DigiNorm) with khmer (0.8.2). TrinityNorm and DigiNorm were ran on the same computer (Intel Core i7-3820 processor). Normalization algorithms were designed to systematize the coverage in shotgun sequencing data sets, thereby removing redundant reads. As a consequence, computational requirements are reduced, supposedly without negatively impacting assembly quality. For TrinityNorm the default commands were run with a max coverage (max_cov) of 30. Before running DigiNorm, reads were shuffled and a kmer length of 25 and coverage of 30 were specified. We trimmed off likely erroneous k-mers with the filter-abund.py script. For each assembly we tracked the runtimes.

### Gene annotation and GO-terms assignment

BLASTX (e-value cut offs < 1e-10) searches were performed against OMA browser mouse proteins and NCBI non-redundant vertebrate protein databases. Search against OMA browser database is a cost-effective way of gene annotation and allows an upper bound estimation of genes orthologous to those of the mouse among the reconstructed contigs. This database contains most or all exons of a given gene, keeping the number of sequences as low as possible. The longest variant is always retained; shorter variants are also kept if they differ by at least in 10% of their sequence from the longer variants retained.

For the BLASTX analysis we report, i) the number of contigs that overlap the proteins of mouse genes at > 99%, > 90%, > 80% and > 50% of their length; ii) the number of distinct mouse proteins reconstructed by a putatively homologous contig using those cutpoints; and iii) the number of distinct genes found with at least one putatively homologous contig reconstructing the gene CDS at or above those percentages. We also reported, the number of contigs, coding sequences, and genes that had a significant hit (e-value < 1e-10) independently of the alignment proportion. To report the number of genes, contigs annotated as putatively homologs to mouse OMA proteins entries were grouped into the corresponding mouse genes using the oma-ensembl file at OMA-Browser webpage through in-house-scripts (Additional file 4).

The contigs assembled from the Multireads, TrinityNorm and DigiNorm strategies, were annotated for protein domains through the Pfam database using HMMER. An e-value threshold of 1e-2 was set. Before running this analysis we first predicted the exon/intron structure of each contig using the software Augustus [45] trained with *Homo sapiens.* This software has been used extensively for gene prediction (e.g. [46–48]). The GTF files from Augustus output are available upon request. Only the non-overlapping protein domains found were reported on the summary file of BLASTX and Pfam results. A in-house-script was used for this purpose (Additional file 4).

The average number of potential isoforms reconstructed from the Trinity assembly was calculated averaging the times that a particular "comp_XXX" (as given by the ID of Trinity assembled contig) is repeated.

Gene Ontology analysis was done using the DAVID bioinformatics database, using the Benjamini correction of *p* < 0.05 as criterion for enrichment. First, we classify the most common GO-terms from the genes list obtained from the TrinityNorm assembly. Then, we used that gene list as background for analyzing the ontology of the most expressed genes.

### List of most expressed genes

To determine the most expressed genes in the kidneys of *A. olivacea*, we sought for genes that were in common among the 5% most expressed genes in each of the 13 transcriptomes. Despite this being a very conservative approach, it was preferred because it would generate a reliable list of genes.

To this end, we first aligned RNA-Seq reads in a paired end fashion against each reference transcript using the aligner Bowtie [49]. Then, we calculated gene-level TPM values using RSEM (v1.2.4). The results of BLASTX for each transcriptome against mouse OMA browser protein, and the OMA-ensembl corresponding pair were used to specify which transcripts were from the same gene. This program hands reads that map to multiple transcripts avoiding throwing away data and biased estimates without relying on the existence of a reference genome. Finally, bash commands and in-house scripts (Additional file 4) were used to obtain the most expressed genes as described above.

### Availability of supporting data

The sequencing data has been deposited to the Sequence Read Archive database (accession number SRP033780).

### Animal ethics statements

All methods involving *A. olivacea* were carried out in accordance with a protocol reviewed and approved by the Ethics Committee of the Fondo Nacional de Ciencia y Tecnología (FONDECYT, Chile) and the Ethics Committee of the Universidad Austral de Chile (UACh, Chile), as part of the review process for the Fondecyt Research Grant 1110737.

## Electronic supplementary material

Additional file 1: **Sampling localities, assembly metrics, contig distribution and gene annotation.** **Table S1.** Specimen ID numbers, sampling localities, and read lengths before and after trimming. **Table S2.** Descriptive statistics of individual RNA-seq samples and reconstructions. **Table S3.** Distribution of contig sizes and their relative proportions for each assembly protocol. **Table S4a.** Gene annotation and CDS reconstruction using BLASTX to OMA browser mouse protein database. **Table S4b.** Annotation using BLASTX to NCBI non-redundant vertebrate protein database. **Table S5.** Annotation through Pfam database using HMMER. (XLS 20 KB)

Additional file 2: **Summarizing the BLASTX and Pfam results for Multireads, TrinityNorm and DigiNorm strategies.** (ZIP 8 MB)

Additional file 3: **List of the 283 most expressed genes and its GO-terms classification.** **Table S5.** List of 283 genes represented in the 5% most expressed genes of each of the individual samples. **Table S6.** Gene Ontology classification of the 283 most expressed genes. (XLS 70 KB)

Additional file 4: **Containing the in-house-python scripts.** (ZIP 3 KB)

## Abbreviations

GO: Gene ontology
CDS: Coding sequences
TrinityNorm: Trinity *in silico* normalization
DigiNorm: Digital normalization.
